# Serum 25-Hydroxyvitamin D Concentration and Auditory P300 Characteristics in Healthy Young Adults: A Cross-Sectional Study

**DOI:** 10.7759/cureus.114819

**Published:** 2026-08-19

**Authors:** Amit Kumar, Tarun Kumar, Pooja Sakshi, Ravi Shekhar, Kumar Abhishek

**Affiliations:** 1 Department of Physiology, Indira Gandhi Institute of Medical Sciences, Patna, IND; 2 Department of Biochemistry, Indira Gandhi Institute of Medical Sciences, Patna, IND

**Keywords:** 25-hydroxyvitamin d, attention, auditory p300, cognitive processing, cross-sectional study, event-related potential, healthy adults

## Abstract

Background: Vitamin D receptors and metabolizing enzymes are expressed in brain regions involved in attention and memory, but evidence relating serum 25-hydroxyvitamin D (25(OH)D) to objective electrophysiological indices of cognition in healthy young adults is limited. We examined the associations between serum 25(OH)D and auditory P300 latency and amplitude.

Methods: This cross-sectional study prospectively enrolled 40 healthy adults aged 18-40 years: 20 with vitamin D nonsufficiency (≤30 ng/mL) and 20 with sufficiency (>30 ng/mL). Total serum 25(OH)D was measured by quantitative delayed one-step competitive chemiluminescent microparticle immunoassay on the ARCHITECT i2000SR platform (Abbott Diagnostics, Abbott Park, IL). Auditory P300 was recorded at Cz using a two-stimulus oddball paradigm. Prespecified primary analyses used independent-samples t-tests. Secondary analysis used analysis of covariance adjusted for age, sex, and body mass index (BMI). Pearson correlation and multivariable linear regression treated serum 25(OH)D as a continuous exposure in exploratory analyses. Holm-Bonferroni correction was applied separately within the primary, secondary, correlation, and regression analysis families.

Results: The groups were comparable in age, sex distribution, height, weight, and BMI. Primary comparisons were not statistically significant. Mean latency was 326.88 ± 9.75 ms in the nonsufficient group and 321.46 ± 8.06 ms in the sufficient group (mean difference: 5.42 ms, 95% confidence interval (CI): -0.31 to 11.14). Mean amplitude was 5.87 ± 0.69 and 6.17 ± 0.53 µV, respectively (mean difference: -0.294 µV, 95% CI: -0.686 to 0.098). The secondary adjusted latency contrast was nominally significant (adjusted mean difference: 5.94 ms, 95% CI: 0.003-11.88; unadjusted p = 0.0499) but did not remain significant after Holm correction (adjusted p = 0.100). In exploratory analyses, serum 25(OH)D correlated inversely with latency (r = -0.450; unadjusted p = 0.004; Holm-adjusted p = 0.007), and its adjusted regression coefficient was -0.437 ms/ng/mL (95% CI: -0.695 to -0.178; unadjusted p = 0.002; Holm-adjusted p = 0.003). The adjusted amplitude coefficient was not significant (Holm-adjusted p = 0.140).

Conclusions: The prespecified group comparisons did not provide statistically conclusive evidence of different P300 latency or amplitude according to the operational vitamin D threshold. The nominal secondary latency contrast did not remain significant after multiplicity correction. The continuous inverse latency association remained statistically significant after Holm correction but was exploratory and does not establish causality, a neurophysiological cutoff, or a rationale for screening or supplementation. Larger prospective studies are needed.

## Introduction

Vitamin D is a secosteroid hormone with biological actions extending beyond calcium and skeletal homeostasis. Vitamin D receptors and the activating enzyme 1α-hydroxylase are expressed in neurons and glial cells within the cortex, hippocampus, hypothalamus, and other regions relevant to cognition [1,2]. Experimental work suggests that vitamin D may influence neurotrophic signaling, neurotransmitter synthesis, neuronal calcium homeostasis, oxidative defense, and neuroimmune regulation [2-4]. These mechanisms provide biological plausibility for an association between circulating 25-hydroxyvitamin D (25(OH)D), the principal marker of vitamin D status, and cognitive function.

The P300 is a positive event-related potential elicited approximately 300 ms after a task-relevant stimulus, most commonly during an auditory or visual oddball paradigm [5,6]. P300 latency reflects the time required for stimulus classification and evaluation, whereas amplitude is influenced by attentional resource allocation, target probability, task relevance, and context updating [7,8]. Because P300 provides millisecond-level temporal resolution, it can characterize the timing and magnitude of stimulus evaluation independently of a participant's final behavioral score [9]. Conventional cognitive tests integrate multiple processes such as comprehension, motor response, motivation, education, and task strategy and may therefore show ceiling effects in healthy young adults with otherwise preserved cognitive performance. P300 may complement these measures by detecting subtle differences in neural information-processing efficiency before they become apparent on conventional neuropsychological testing. Accordingly, examining P300 in a young, clinically healthy population provides an opportunity to investigate subclinical neurophysiological variation while minimizing the influence of overt cognitive disease and age-related neurodegeneration.

Most human research on vitamin D and cognition has involved older adults, dementia, mild cognitive impairment, or clinical populations. Observational studies have often linked lower 25(OH)D with poorer executive function or a greater risk of cognitive impairment, but residual confounding and reverse causation remain important concerns [10-13]. Smaller trials in adolescents or adults have reported task-specific findings involving planning, problem-solving, or stress resilience [14,15], and a clinical study associated hypovitaminosis D with poorer executive performance [16]. Randomized evidence remains inconsistent: systematic reviews have found mixed or modest effects, and a recent trial found no cognitive improvement among older adults with mild-to-moderate vitamin D deficiency [17-20].

Evidence directly linking vitamin D status with P300 characteristics is particularly sparse. In the Menopause Analytical Hormonal Correlate Outcome Study, vitamin D measures clustered with P300 latency in exploratory factor analysis, but vitamin D did not remain an independent predictor after correction for multiple hormonal variables [21]. Whether a similar relationship is detectable in healthy young adults, in whom age-related neurodegeneration and major clinical comorbidity are minimized, remains uncertain.

The primary objective of this study was to compare auditory P300 latency and amplitude between healthy adults in two prespecified vitamin D categories: nonsufficient and sufficient. Additional exploratory objectives were to evaluate serum 25(OH)D continuously in relation to P300 outcomes and to assess whether any association persisted after adjustment for age, sex, and body mass index (BMI). We hypothesized that the nonsufficient group would have longer P300 latency and lower P300 amplitude.

## Materials and methods

Study design and setting

This cross-sectional observational neurophysiological study used prospective recruitment from May 2024 to October 2025 in the Neurophysiology Laboratory, Department of Physiology, Indira Gandhi Institute of Medical Sciences (IGIMS), Patna, India, in collaboration with the Department of Biochemistry. The Institutional Ethics Committee of IGIMS approved the protocol (approval number 1539/IEC/IGIMS/2024). Written informed consent was obtained from every participant, and the study was conducted in accordance with the Declaration of Helsinki.

Sampling and sample size

Participants were recruited using convenience sampling from healthy adult attendants accompanying patients to different departments of IGIMS. Recruitment incorporated prespecified equal vitamin D-category quotas: serum 25(OH)D was measured after enrollment, and eligible participants were classified as vitamin D nonsufficient (≤30 ng/mL) or sufficient (>30 ng/mL), with recruitment continuing until 20 participants had been included in each category. Potential participants were approached among adult attendants present in participating hospital departments and invited to undergo an eligibility assessment and serum 25(OH)D testing. Enrollment was convenience-based rather than population-based; after biochemical classification, eligible participants were retained while the corresponding prespecified vitamin D-category quota remained open. Thus, the final 1:1 distribution of vitamin D categories was imposed by the sampling design rather than reflecting the prevalence of vitamin D nonsufficiency in the source population. P300 recording was subsequently performed in all 40 participants, and group classification was determined solely by serum 25(OH)D concentration. A comprehensive screening log documenting every individual approached but not enrolled was not maintained; therefore, the total numbers approached and excluded before final enrollment cannot be reconstructed reliably.

The sample-size estimate was informed by Grung et al. [14], the closest available study relating vitamin D status to executive-function performance. Because no directly transferable correlation coefficient or P300-specific between-group effect estimate was available, a moderate anticipated correlation of r = 0.45 was adopted as a protocol planning assumption. The sample size for testing a single correlation was estimated using Fisher's z-transformation:

$n=\left[\frac{Z_{1-\alpha/2}+Z_{1-\beta}}{\frac{1}{2}\ln\left(\frac{1+r}{1-r}\right)}\right]^2+3$

With a two-sided α of 0.05 (Z_₁₋α/₂_ = 1.96), 80% power (Z_₁₋β_ = 0.84), and r = 0.45, the minimum sample was approximately 37 participants. Allowing about 8% for incomplete data or technically unacceptable recordings increased the target to 40. The protocol specified equal allocation of 20 participants to each vitamin D category for the primary group comparisons. Because this calculation was based on a continuous association rather than a P300 group contrast, the categorical analyses may have had limited power to detect smaller differences. Because the primary outcomes were ultimately analyzed as between-group comparisons, we additionally evaluated the precision implied by the prespecified allocation. With 20 participants per group, a two-sided α of 0.05, and equal group sizes, the study provided approximately 80% power to detect a standardized mean difference of about 0.91 between vitamin D categories. Therefore, the primary categorical analysis was adequately powered only for relatively large between-group effects and may have lacked power to detect smaller or moderate differences. This limitation is considered when interpreting the nonsignificant primary results.

Participants and eligibility criteria

Healthy men and women aged 18-40 years were assessed for eligibility. The criteria applied before biochemical sampling and electrophysiological recording are summarized in Table 1.

**Table 1:** Eligibility criteria Participants were required to meet all inclusion criteria and none of the exclusion criteria. P300 is the event-related potential

Criterion type	Criterion
Inclusion	Age 18-40 years
	Male or female sex
	Written informed consent provided
Exclusion	Refusal or inability to provide written informed consent
	Diabetes mellitus, dyslipidemia, or thyroid dysfunction
	History of stroke, epilepsy, Parkinson disease, or another major neurological or neurodegenerative disorder
	Known hearing impairment or auditory pathway disorder likely to interfere with auditory P300 testing
	Current use of medication known to affect cognitive function
	Known allergy to electrode gel or adhesive materials
	Chronic illness or acute systemic disease at the time of assessment

Prerecording standardization and anthropometry

All P300 recordings were conducted between 10:00 a.m. and 1:00 p.m. to minimize variation due to testing time. Participants were asked to avoid strenuous physical activity for 48 hours and caffeine, tea, nicotine, and alcohol for 24 hours before testing. Recordings were performed at least two hours after a light meal. Testing was conducted individually in a quiet, dimly lit, sound-attenuated laboratory maintained at approximately 25°C ± 2°C. Age and sex were recorded, height and weight were measured using standard techniques, and BMI was calculated as weight in kilograms divided by height in meters squared.

P300 event-related potential recording

Auditory P300 was recorded using a four-channel Neuro-MEP-Micro electrophysiology system (Neurosoft, Ivanovo, Russia; software version 2009). After scalp preparation, silver/silver-chloride electrodes were positioned according to the International 10-20 system, with the active electrode at Cz, linked mastoids as the reference, and Fpz as ground. Electrode impedance was maintained below 5 kΩ.

A two-stimulus auditory oddball paradigm was delivered binaurally through headphones. Frequent 1,000-Hz standard tones constituted approximately 70% of stimuli, and rare 2,000-Hz target tones constituted approximately 30%. Stimuli were presented at an 85 dB sound pressure level, with a duration of 100 ms and a stimulation frequency of 1 Hz; random variation in the interstimulus period was enabled. Participants attended to the target tones and responded by button press. Signals were acquired at 1,000 Hz using an amplifier input range of 1.5 mV, with a 0.5-Hz high-pass filter, a 35-Hz low-pass filter, a 50-Hz notch filter, and a high-harmonics filter enabled. Automatic artifact rejection was applied at 300 µV, weighted averaging was used, and up to 100 stimuli were delivered to obtain approximately 30 accepted target and 70 accepted standard averages. P300 latency was identified as the largest positive deflection within 250-450 ms, and amplitude was measured from the prestimulus baseline to the positive peak. Peaks identified by the software were manually verified by an assessor blinded to participants' vitamin D concentration and group allocation. Latency and amplitude were expressed in milliseconds and microvolts, respectively [5,7,9]. Each participant contributed one standardized recording session for the present analysis; formal within-session or test-retest repeatability coefficients were not estimated. Quality control relied on impedance control, automated artifact rejection, acquisition of approximately 30 accepted target and 70 accepted standard averages, predefined latency-window identification, and blinded manual verification of software-identified peaks. Formal audiometry was not performed; eligibility relied on the absence of known hearing impairment or auditory pathway disease.

Serum 25(OH)D estimation

Five milliliters of venous blood was collected under aseptic precautions. Samples were centrifuged at 3,000 rpm for 10 minutes. Total serum 25(OH)D was measured in the Department of Biochemistry, IGIMS, using a quantitative delayed one-step competitive immunoassay based on chemiluminescent microparticle immunoassay technology on the Abbott ARCHITECT i2000SR analyzer (Abbott Diagnostics, Abbott Park, IL). Concentrations were expressed in nanograms per milliliter.

For the primary categorical analyses, the deficiency and insufficiency strata in the parent protocol were combined into a nonsufficient group (≤30 ng/mL), whereas concentrations >30 ng/mL were classified as sufficient. This operational classification was informed by the historical 2011 Endocrine Society framework, which defined deficiency as <20 ng/mL and insufficiency as 21-29 ng/mL [22]. The 2024 Endocrine Society guideline emphasizes that outcome-specific serum 25(OH)D thresholds have not been established for generally healthy adults [23]. The 30-ng/mL threshold was therefore a prespecified study classification and not a validated cognitive, electrophysiological, or clinical cutoff.

Outcomes

The primary outcomes were P300 latency and P300 amplitude. The primary exposure was vitamin D status categorized as nonsufficient or sufficient. Serum 25(OH)D was additionally analyzed as a continuous exposure in exploratory association analyses. Age, sex, and BMI were prespecified covariates because they may influence vitamin D status, P300 characteristics, or both.

Statistical analysis

Data were analyzed using IBM Statistical Package for the Social Sciences Statistics for Windows, version 26.0 (IBM Corp., Armonk, NY). Continuous variables were summarized as mean ± standard deviation, and categorical variables as number and percentage. Distributional assumptions were assessed using the Shapiro-Wilk test, histograms, and normal Q-Q plots. Baseline continuous variables were compared with independent-samples t-tests; Welch's t-test was used when variance homogeneity was not satisfied. Sex distribution was compared with Pearson's chi-square test, with Fisher's exact test also reported for the 2 × 2 table. P300 latency and amplitude, and their comparison across the prespecified vitamin D categories, were defined as the primary outcomes in the study protocol. The study was not prospectively registered with a public statistical-analysis-plan repository; secondary analysis of covariance (ANCOVA) and continuous association analyses are therefore identified separately and interpreted cautiously, with the latter treated as exploratory.

**Figure 1:**
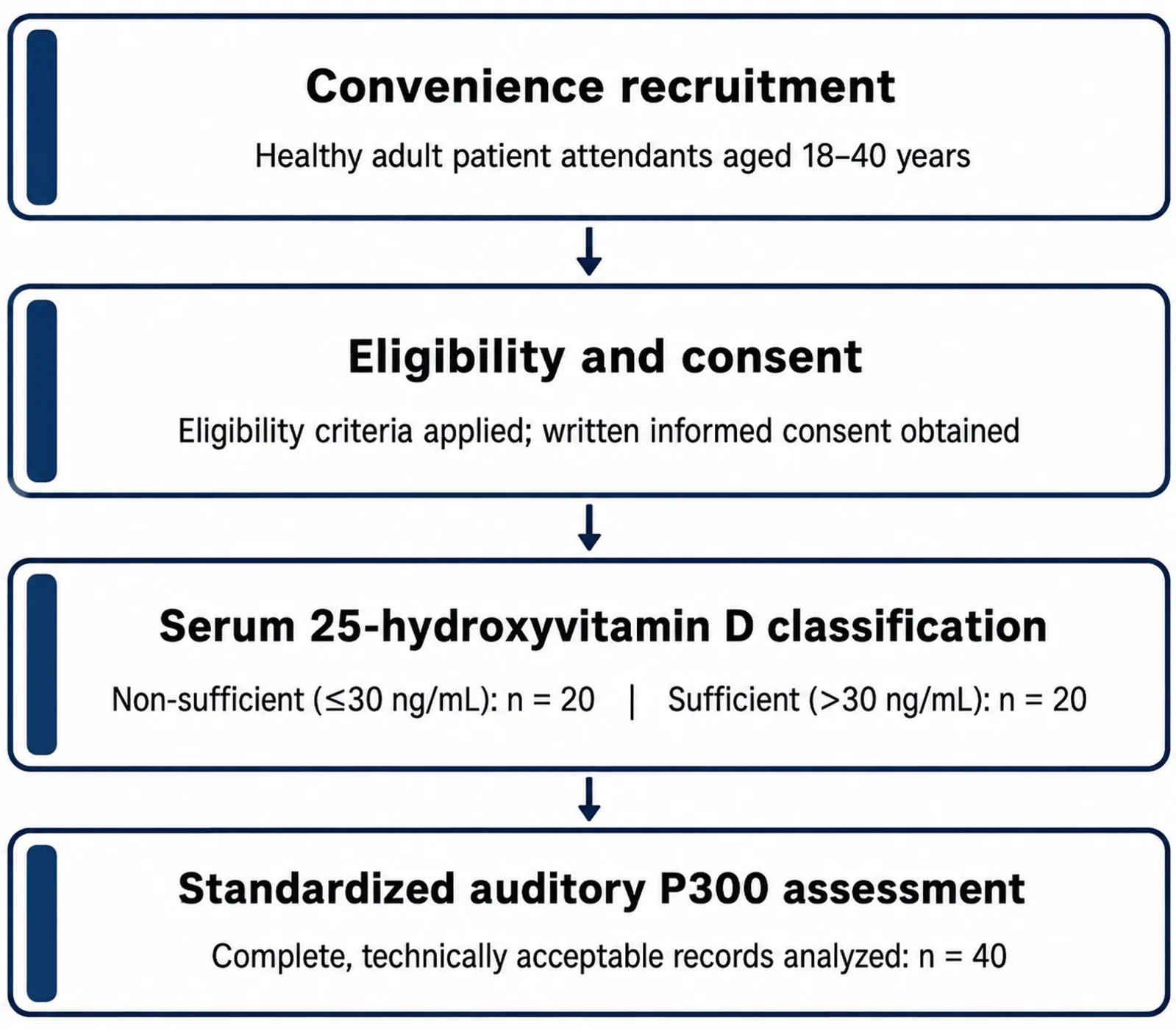
Participant recruitment and study workflow Recruitment continued until 20 eligible participants were included in each prespecified vitamin D category. All 40 complete, technically acceptable records were analyzed. P300 is the event-related potential

The prespecified primary analyses compared P300 latency and amplitude between vitamin D groups using independent-samples t-tests, with mean differences, 95% confidence intervals (CIs), and Hedges' g. Secondary ANCOVA estimated group differences adjusted for age, sex, and BMI, with partial eta-squared (partial η²). Homogeneity of regression slopes was evaluated using group-by-covariate interaction terms. Additional exploratory analyses evaluated continuous serum 25(OH)D using Pearson correlation and separate multivariable linear regression models for latency and amplitude adjusted for age, sex, and BMI. Sex was coded female = 0 and male = 1. Regression diagnostics included residual distribution, residual vs. predicted plots, Cook's distance, tolerance, and variance inflation factors (VIFs). Visual inspection of residual vs. predicted plots was used to assess linearity and homoscedasticity. All tests were two-sided, and p < 0.05 was the nominal significance threshold. To account for testing of both P300 outcomes, the Holm-Bonferroni procedure was applied separately within each prespecified analysis family: primary independent-samples t-tests, secondary ANCOVA models, exploratory Pearson correlations, and exploratory vitamin D regression coefficients. Both unadjusted and Holm-adjusted p values are reported. The recruitment, classification, and assessment sequence is summarized in Figure 1.

## Results

All 40 participants had complete data for the variables in the present analysis. P300 latency and amplitude did not depart significantly from normality in either vitamin D group: latency, W = 0.948, p = 0.337 in the nonsufficient group and W = 0.984, p = 0.975 in the sufficient group; amplitude, W = 0.959, p = 0.522 and W = 0.979, p = 0.926, respectively. The primary analyses therefore used independent-samples t-tests, followed by secondary ANCOVA adjusted for age, sex, and BMI.

Participant characteristics

The analytic cohort comprised 20 participants in each vitamin D category. Twenty-five (62.5%) were female, and 15 (37.5%) were male participants. No missing or implausible values were identified. Age, sex distribution, height, weight, and BMI were comparable between groups. Serum 25(OH)D differed, as expected, from the exposure-based group definition (as shown in Table 2).

**Table 2:** Demographic, anthropometric, and biochemical characteristics by vitamin D category Values are mean ± SD unless otherwise stated. Mean differences were calculated as nonsufficient minus sufficient. Sex distribution was tested using a 2 × 2 table; Fisher's exact test two-sided p = 0.514 and Cramer's V = 0.155 BMI: body mass index; CI: confidence interval; MD: mean difference; SD: standard deviation; 25(OH)D: 25-hydroxyvitamin D

Variable	Nonsufficient (n = 20)	Sufficient (n = 20)	Estimate (95% CI)	Statistic	p value
Age, years	26.97 ± 5.31	26.82 ± 5.41	MD: 0.16 (-3.28 to 3.59)	t = 0.091	0.928
Sex, n (%)					
Female	14 (70.0)	11 (55.0)	-	χ² = 0.960	0.327
Male	6 (30.0)	9 (45.0)			
Height, cm	167.05 ± 5.62	167.75 ± 6.63	MD: -0.70 (-4.64 to 3.24)	t = -0.360	0.721
Weight, kg	63.71 ± 9.29	65.45 ± 9.93	MD: -1.75 (-7.90 to 4.41)	t = -0.574	0.569
BMI, kg/m²	22.82 ± 2.99	23.19 ± 2.62	MD: -0.38 (-2.17 to 1.42)	t = -0.422	0.675
Serum 25(OH)D, ng/mL	18.65 ± 10.01	34.80 ± 4.44	MD: -16.15 (-21.18 to -11.12)	Welch t = -6.593	<0.001

P300 characteristics by vitamin D category

The nonsufficient group had a longer mean P300 latency than the sufficient group (326.88 ± 9.75 vs. 321.46 ± 8.06 ms; mean difference 5.42 ms, 95% CI: -0.31 to 11.14), although the difference was not statistically significant after Holm correction (unadjusted p = 0.063; Holm-adjusted p = 0.126; Hedges’ g = 0.594) (as shown in Table 3). Mean P300 amplitude was lower in the nonsufficient group than in the sufficient group (5.87 ± 0.69 vs. 6.17 ± 0.53 µV; mean difference: -0.294 µV, 95% CI: -0.686 to 0.098), but the difference was not statistically significant (unadjusted and Holm-adjusted p = 0.137; Hedges’ g = -0.470) (as shown in Table 3).

**Table 3:** Unadjusted and adjusted comparisons of P300 outcomes between vitamin D categories Values for t-tests are mean ± SD; ANCOVA group values are adjusted estimated marginal means after controlling for age, sex, and BMI. Differences were calculated as nonsufficient minus sufficient. The t statistic has 38 degrees of freedom, and the ANCOVA F statistic has 1 and 35 degrees of freedom. Holm-Bonferroni correction was applied separately to the two primary t-tests and to the two secondary ANCOVA outcomes. For t-tests, the effect size is Hedges' g; for ANCOVA, it is partial η² ANCOVA: analysis of covariance; BMI: body mass index; CI: confidence interval; SD: standard deviation

Outcome	Analysis	Nonsufficient	Sufficient	Difference (95% CI)	Statistic	Unadjusted p value	Holm-adjusted p value	Effect size
Latency, ms	t-test	326.88 ± 9.75	321.46 ± 8.06	5.42 (-0.31 to 11.14)	t = 1.916	0.063	0.126	g = 0.594
	Adjusted ANCOVA	327.46	321.51	5.94 (0.003 to 11.88)	F = 4.126	0.0499	0.100	η² = 0.105
Amplitude, µV	t-test	5.87 ± 0.69	6.17 ± 0.53	-0.294 (-0.686 to 0.098)	t = -1.518	0.137	0.137	g = -0.470
	Adjusted ANCOVA	5.910	6.161	-0.251 (-0.636 to 0.133)	F = 1.765	0.193	0.193	η² = 0.048

In secondary ANCOVA, the adjusted latency contrast had a small positive lower confidence bound (unadjusted p = 0.0499), but it did not remain statistically significant after Holm correction (adjusted p = 0.100). The adjusted amplitude contrast remained nonsignificant (unadjusted and Holm-adjusted p = 0.193). Group-by-age, group-by-BMI, and group-by-sex interaction terms were nonsignificant for both outcomes, supporting the homogeneity-of-regression-slopes assumption (as shown in Table 3).

Exploratory continuous associations of serum 25(OH)D with P300

**Figure 2:**
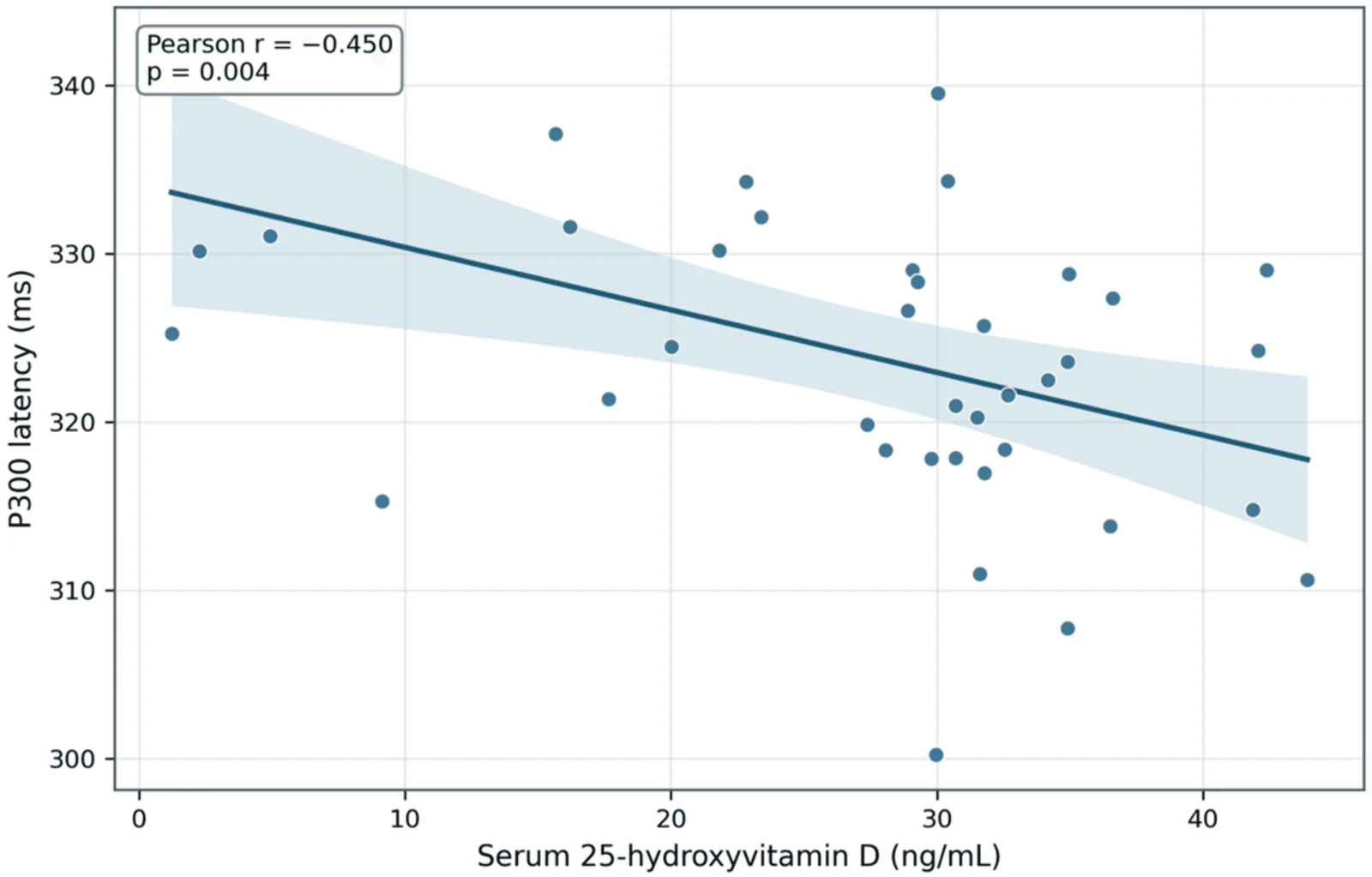
Association between serum 25-hydroxyvitamin D concentration and P300 latency Each point represents one participant. The line is the unadjusted fitted linear regression and the shaded region is its 95% confidence band. Pearson r = −0.450; unadjusted p = 0.004; Holm-adjusted p = 0.007.

**Figure 3:**
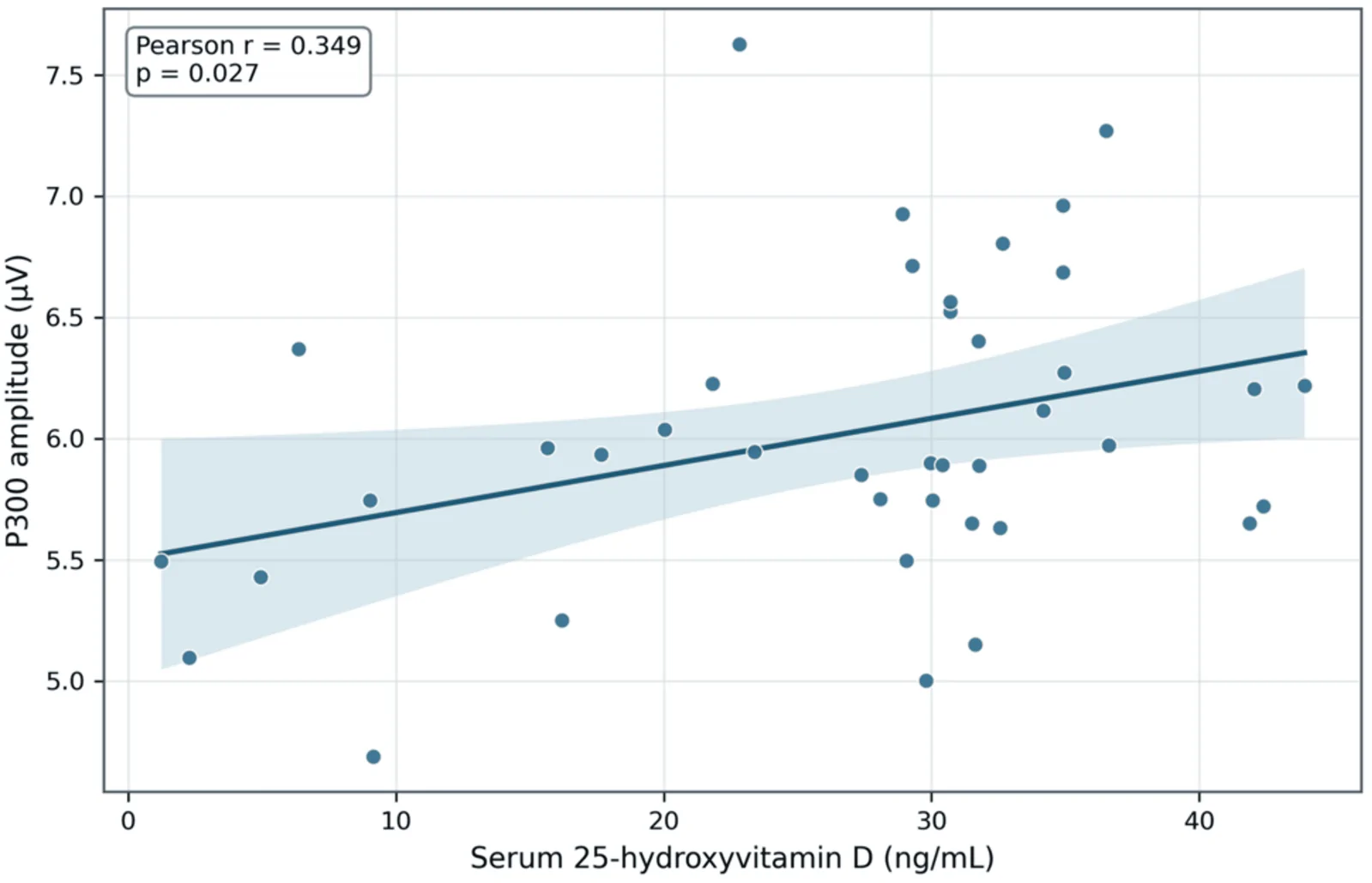
Association between serum 25-hydroxyvitamin D concentration and P300 amplitude Each point represents one participant. The line is the unadjusted fitted linear regression and the shaded region is its 95% confidence band. Pearson r = 0.349; unadjusted p = 0.027; Holm-adjusted p = 0.027.

After completion of the prespecified categorical comparisons, additional exploratory analyses examined serum 25(OH)D as a continuous exposure. These analyses were secondary to, and do not supersede, the nonsignificant primary group comparisons. Serum 25(OH)D showed a moderate inverse Pearson correlation with P300 latency and a weaker positive correlation with P300 amplitude (Table 4; Figures 2, 3). Both associations remained statistically significant after Holm correction within the two-outcome correlation family, but they require independent confirmation because the study was small, cross-sectional, and based on quota recruitment into equal vitamin D categories.

**Table 4:** Correlations of serum 25-hydroxyvitamin D concentration with P300 outcomes Pearson product-moment correlations were exploratory. Holm-Bonferroni correction was applied across the two P300 outcomes CI: confidence interval

Outcome	Pearson r	95% CI	Unadjusted p value	Holm-adjusted p value
P300 latency	-0.450	-0.668 to -0.161	0.004	0.007
P300 amplitude	0.349	0.042 to 0.596	0.027	0.027

Exploratory multivariable analyses

In the exploratory multivariable analysis, higher serum 25(OH)D remained associated with shorter P300 latency after adjustment for age, sex, and BMI. The latency model accounted for 27.1% of the observed variance (model p = 0.023). The vitamin D coefficient remained statistically significant after Holm correction within the two-outcome regression family (unadjusted p = 0.002; Holm-adjusted p = 0.003). The amplitude model was not statistically significant overall (model p = 0.080), and the adjusted serum 25(OH)D coefficient for amplitude was also nonsignificant (unadjusted and Holm-adjusted p = 0.140). VIF values ranged from 1.030 to 1.141. Visual inspection of standardized residual vs. predicted value plots showed no obvious curvature or funnel-shaped pattern. Standardized residuals remained within ±3. Maximum Cook's distance was 0.215 for latency and 0.186 for amplitude, indicating that no single observation exerted excessive influence by conventional criteria (as shown in Table 5).

**Table 5:** Multivariable linear regression models for P300 latency and amplitude Female sex was the reference category. Holm-Bonferroni correction was applied to the serum 25(OH)D coefficients across the two regression outcomes. Latency model: R² = 0.271, adjusted R² = 0.188, F(4, 35) = 3.255, model p = 0.023. Amplitude model: R² = 0.207, adjusted R² = 0.116, F(4, 35) = 2.286, model p = 0.080 B: unstandardized coefficient; BMI: body mass index; CI: confidence interval; SE: standard error; VIF: variance inflation factor; 25(OH)D: 25-hydroxyvitamin D

Outcome	Predictor	B (95% CI)	SE	Standardized beta	t	Unadjusted p value	Holm-adjusted p value	VIF
Latency	Serum 25(OH)D, ng/mL	-0.437 (-0.695 to -0.178)	0.127	-0.529	-3.432	0.002	0.003	1.141
	Age, years	-0.098 (-0.618 to 0.421)	0.256	-0.056	-0.384	0.703	-	1.030
	Male vs. female	3.219 (-2.414 to 8.851)	2.774	0.171	1.160	0.254	-	1.039
	BMI, kg/m^2^	0.710 (-0.313 to 1.733)	0.504	0.213	1.409	0.168	-	1.101
Amplitude	Serum 25(OH)D, ng/mL	0.013 (-0.005 to 0.032)	0.009	0.242	1.508	0.140	0.140	1.141
	Age, years	0.017 (-0.019 to 0.054)	0.018	0.146	0.953	0.347	-	1.030
	Male vs. female	0.123 (-0.273 to 0.519)	0.195	0.097	0.631	0.532	-	1.039
	BMI, kg/m^2^	0.057 (-0.015 to 0.129)	0.035	0.255	1.614	0.116	-	1.101

## Discussion

The prespecified unadjusted comparisons did not show statistically significant differences in P300 latency or amplitude between the vitamin D categories, and both remained nonsignificant after Holm correction. Although the nonsufficient group had a mean latency approximately 5.4 ms longer and an amplitude approximately 0.29 µV lower, the 95% CIs for both primary mean differences included zero. Secondary ANCOVA produced a nominal latency contrast (unadjusted p = 0.0499) with a lower confidence bound close to zero, but this finding did not remain significant after Holm correction (adjusted p = 0.100); adjusted amplitude remained nonsignificant. The categorical findings are statistically inconclusive and should not be interpreted as evidence of equivalence. Exploratory continuous analyses showed an inverse association with latency that remained significant after correction but requires confirmation in an independent cohort. Accordingly, the principal finding of this study is the absence of statistically conclusive evidence for differences in either prespecified P300 outcome between the two vitamin D categories.

The nonsignificant primary comparisons are compatible with the limited and inconsistent literature on vitamin D and neurocognitive electrophysiology. Braverman et al. examined hormonal and metabolic correlates of P300 in a large clinical cohort of women and found that vitamin D measures clustered with P300 latency in factor analysis, although vitamin D did not remain an independent predictor after correction for multiple hormonal variables [21]. Differences in age, sex, menopausal status, comorbidity, exposure definition, and analytical strategy limit direct comparison. Our findings add evidence from a younger healthy population but do not establish that a conventional vitamin D threshold corresponds to a discrete electrophysiological phenotype.

The inverse association between continuous 25(OH)D and latency is directionally consistent with observational evidence relating lower vitamin D status to poorer executive function or psychomotor speed. Kueider et al. reported associations of lower 25(OH)D and vitamin D-related genetic markers with poorer executive function and psychomotor speed in the Baltimore Longitudinal Study of Aging [10]. Grung et al. and Hansen et al. reported task-specific findings involving planning or problem-solving [14,15], whereas Falasca et al. observed poorer executive performance with hypovitaminosis D in adults with HIV [16]. These studies used different populations and outcomes, and directional consistency with the present exploratory correlation is not evidence of causality.

Intervention evidence is also inconsistent. A review of 20 randomized trials found mixed results without consistent support for a cognitive benefit of vitamin D supplementation in adults [17]. A systematic review restricted to healthy individuals reported possible improvement in selected domains but emphasized heterogeneity and the small number of eligible trials [18]. A 2024 meta-analysis identified only a small overall effect and no consistent benefit across specific domains [19]. The VitaMIND randomized controlled trial subsequently found no measurable cognitive improvement in older adults with mild-to-moderate deficiency [20]. Conversely, selected trials in older adults with mild cognitive impairment or in community-dwelling women reported improvements or preservation in certain cognitive measures [24,25]. Thus, an observational association with P300 cannot be translated into an expectation that supplementation will improve cognition.

Potential neurophysiological interpretation

Several mechanisms could plausibly connect vitamin D status with P300 timing. Vitamin D signaling is present in cortical and hippocampal neurons and may regulate neurotrophins, neurotransmitter systems, calcium-channel expression, antioxidant defenses, and inflammatory pathways [1-4]. More efficient synaptic signaling or better preservation of neuronal excitability could theoretically shorten stimulus-evaluation time. These mechanisms were not measured in the present study and remain hypotheses rather than demonstrated mediators.

The differing latency and amplitude patterns should also be interpreted cautiously. P300 latency is a temporal marker of stimulus evaluation, whereas amplitude is influenced by arousal, target probability, attentional engagement, reference montage, trial count, and signal-to-noise ratio [7-9]. These factors can increase amplitude variability. Nevertheless, the nonsignificant primary comparisons mean that mechanistic explanations remain speculative. The latency association may reflect a continuous biological gradient, residual confounding, quota-based sampling, or chance.

Primary categorical findings and exploratory continuous analyses

The categorical and continuous analyses addressed different questions. The primary categorical comparisons did not provide statistically conclusive evidence of differences in P300 latency or amplitude, including after Holm correction. Exploratory continuous analyses retained more information and identified a latency association that remained significant after correction, but they do not supersede the primary results. Categorizing a continuous biomarker reduces information and assumes a step change at the selected threshold. The >30-ng/mL boundary originated from a historical guideline framework [22], whereas current guidance does not establish a 25(OH)D threshold for cognitive or electrophysiological outcomes in generally healthy adults [23]. The study threshold should therefore be regarded as operational rather than as a validated neurophysiological cutoff.

Strengths and limitations

This study used P300 as an objective, temporally precise index of information processing. Acquisition was standardized by controlled pretest instructions, a fixed auditory oddball paradigm, low electrode impedance, automated artifact rejection, and a defined peak window. Software-identified peaks were manually verified by an assessor blinded to vitamin D concentration and group allocation. Serum 25(OH)D was measured in the same institutional setting using the Abbott ARCHITECT i2000SR platform. Continuous and categorical results were reported with effect estimates and 95% CIs; adjusted analyses considered age, sex, and BMI, and multiplicity was controlled within prespecified analysis families using the Holm-Bonferroni procedure.

Several limitations are important. First, the cross-sectional design precludes conclusions about temporality or causality. Vitamin D concentration may mark differences in outdoor activity, diet, sleep, physical fitness, socioeconomic conditions, or other health behaviors rather than cause altered electrophysiology. Second, the sample was modest. Sample-size planning used an anticipated continuous association rather than a P300-specific group effect, so the primary categorical comparisons may have been underpowered for smaller differences. The exploratory regression models included four predictors in only 40 participants and may yield unstable estimates. Nonsignificant findings are not evidence of equivalence. Third, participants were recruited by single-center convenience sampling with prespecified equal vitamin D-category quotas. Consequently, the observed 1:1 distribution of nonsufficient and sufficient participants was imposed by design and cannot estimate the prevalence of vitamin D nonsufficiency in the underlying population. Because recruitment was deliberately continued until equal numbers were obtained on either side of the prespecified 30-ng/mL threshold, the serum 25(OH)D distribution in the analyzed cohort was shaped by the sampling design rather than arising naturally from an unselected population. Such exposure-based quota sampling can alter the range, density, and relative representation of 25(OH)D values. Because a correlation coefficient depends in part on the distribution and variance of the exposure, this design could either magnify or attenuate the observed continuous association and may also affect the regression slope. The exploratory correlation and regression estimates therefore should not be assumed to represent the magnitude of association that would be observed in a randomly or consecutively sampled community cohort. This sampling strategy also limits external validity because the resulting cohort may differ systematically from healthy young adults in the wider community. Fourth, season, sunlight exposure, dietary vitamin D, supplementation, education, sleep duration, mood, and physical activity were not available for adjustment. Fifth, although Holm-Bonferroni correction was applied separately within prespecified primary, secondary, correlation, and regression families, the number of analytical approaches and modest sample size still increase the possibility of unstable or chance findings. Serum 25(OH)D was measured once and may not represent long-term status; concentrations can also vary modestly across immunoassay platforms. In addition, because a comprehensive screening log was not maintained, the total number of individuals approached or excluded before final enrollment cannot be reported. Finally, formal audiometry was not performed, P300 was analyzed at a single central electrode, and amplitude may be sensitive to reference montage and state-related variability.

Clinical and research implications

The findings should be viewed as statistically inconclusive categorical comparisons accompanied by a hypothesis-generating continuous latency association that remained significant after Holm correction. They do not establish a diagnostic cutoff, justify population screening, or support vitamin D supplementation for cognitive enhancement. Future studies should prospectively power the primary group contrast, document relevant environmental and behavioral covariates, and replicate P300 recordings. Larger multicenter cohorts and interventional studies are required before clinical interpretation.

## Conclusions

Among healthy adults aged 18-40 years, the prespecified categorical comparisons did not provide statistically conclusive evidence of differences in P300 latency or amplitude between vitamin D nonsufficient and sufficient participants, even after Holm correction. A nominal secondary adjusted latency contrast did not remain significant after correction. An exploratory inverse association between continuous serum 25(OH)D and P300 latency remained statistically significant after correction, but it does not establish causality or a neurophysiological threshold. These findings are hypothesis-generating and require confirmation in larger, prospectively designed studies.
